# Laboratory-Evolved Mutants of an Exogenous Global Regulator, IrrE from *Deinococcus radiodurans*, Enhance Stress Tolerances of *Escherichia coli*

**DOI:** 10.1371/journal.pone.0016228

**Published:** 2011-01-18

**Authors:** Tingjian Chen, Jianqing Wang, Rong Yang, Jicong Li, Min Lin, Zhanglin Lin

**Affiliations:** 1 Department of Chemical Engineering, Tsinghua University, Beijing, China; 2 Key Laboratory of Crop Biotechnology, Ministry of Agriculture, Biotechnology Research Institute, Chinese Academy of Agricultural Sciences, Beijing, China; University of California Merced, United States of America

## Abstract

**Background:**

The tolerance of cells toward different stresses is very important for industrial strains of microbes, but difficult to improve by the manipulation of single genes. Traditional methods for enhancing cellular tolerances are inefficient and time-consuming. Recently, approaches employing global transcriptional or translational engineering methods have been increasingly explored. We found that an exogenous global regulator, irrE from an extremely radiation-resistant bacterium, *Deinococcus radiodurans*, has the potential to act as a global regulator in *Escherichia coli*, and that laboratory-evolution might be applied to alter this regulator to elicit different phenotypes for *E. coli*.

**Methodology/Principal Findings:**

To extend the methodology for strain improvement and to obtain higher tolerances toward different stresses, we here describe an approach of engineering *irrE* gene in *E. coli*. An irrE library was constructed by randomly mutating the gene, and this library was then selected for tolerance to ethanol, butanol and acetate stresses. Several mutants showing significant tolerances were obtained and characterized. The tolerances of *E. coli* cells containing these mutants were enhanced 2 to 50-fold, based on cell growth tests using different concentrations of alcohols or acetate, and enhanced 10 to 100-fold based on ethanol or butanol shock experiments. Intracellular reactive oxygen species (ROS) assays showed that intracellular ROS levels were sharply reduced for cells containing the *irrE* mutants. Sequence analysis of the mutants revealed that the mutations distribute cross all three domains of the protein.

**Conclusions:**

To our knowledge, this is the first time that an exogenous global regulator has been artificially evolved to suit its new host. The successes suggest the possibility of improving tolerances of industrial strains by introducing and engineering exogenous global regulators, such as those from extremophiles. This new approach can be applied alone or in combination with other global methods, such as global transcriptional machinery engineering (gTME) for strain improvements.

## Introduction

In recent years, more and more attention has been attached to the production of fuels and chemicals through native or engineered microbes [1], [2], and synthetic biology has accelerated the development of industrial strains [3], [4]. Tolerance of these microorganisms toward stresses (including harmful substrates, toxic contaminants, metabolic products and byproducts at high concentrations) is often crucial for the economics of a bioprocess. However, tolerances are complex phenotypes determined by multiple genes [5]. Present methods used for the improvement of strain tolerance include traditional chemical and physical mutagenesis followed by selection under challenging conditions [6], genome shuffling [7], [8], reprogramming of gene transcription using artificial transcription factors [9], [10], and introduction or over-expression of proteins of specific functions, such as heat shock proteins (HSPs), or enzymes related to the production of protective substances or stress signal transduction [11], [12]. Recently global transcriptional machinery engineering (gTME) has been widely explored to improve strain phenotypes by engineering transcription factors or RNA polymerase subunits, thereby altering transcriptional profiles of target strains; various degrees of success have been achieved [13], [14], [15].

Recently, we found that when an exogenous global regulator (DR0167), *irrE*, from *Deinococcus radiodurans*, was over-expressed in *E. coli* JM109, the cellular tolerances toward osmotic stress, heat stress and oxidative stress were improved. It has also been found that when *irrE* was introduced into *E. coli* cells and constitutively expressed under the control of the GroESL promoter, the irradiation tolerance of *E. coli* cells increased [16]. Extremophiles grow and evolve in extremely tough environments, and are thus pools of stress-resistance genes [17], [18], [19]. *Deinococcus radiodurans*, a bacterium which has received extensive attention for many years, can survive a very high level of ionizing radiation [20], and *irrE*, also named *pprI*, was found to be a crucial switch for the extreme radio resistance of *D. radiodurans*. The expression of *recA* and *pprA* and the activity of catalases in *D. radiodurans* were found to be related to the expression of *irrE* [21], [22]. To explore the mechanism whereby *irrE* confers osmotic tolerance to *E. coli*, we performed proteomic analysis of *E. coli* cells harboring *irrE*, and we found that the protein expression profile of the strain harboring *irrE* was very different from that of the control strain when challenged by NaCl [23]. In total, 124 proteins were differentially expressed, which is comparable with the number of genes whose expression is altered in the gTME approach. For example, the application of gTME in *E. coli* for enhanced ethanol tolerance, produced mutants in which 125, 82 and 72 genes were differentially expressed due to mutated sigma factors in three sequential rounds of evolution, respectively [14].

However, when we tested the ethanol tolerance for *irrE*-harboring *E. coli* cells, no obvious improvement was observed. We subsequently went on to randomly mutate the regulator gene in the hope that genetic mutations would elicit different global profiles of gene transcription and protein expression, and thus different tolerance traits. Upon bench evolution of *irrE*, we indeed obtained several improved mutants with much higher ethanol tolerance compared with cells harboring wild-type *irrE*. We then extended this approach for two other important stresses, butanol and acetate, and again produced improved mutants with higher tolerances toward these stresses. This suggests that *irrE* might become an evolvable tolerance-enhancer for *E. coli* and perhaps other microorganisms as well.

## Results

### Selection and characterization of ethanol-tolerant mutants

**Figure 1:**
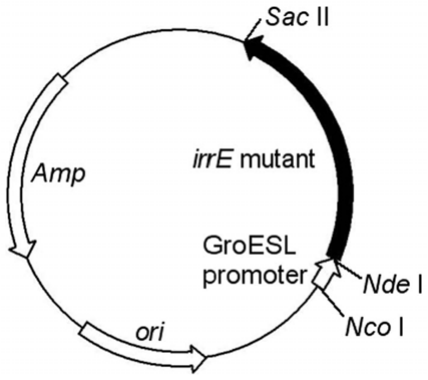
Plasmid map for *irrE* library construction and expression of *irrE* mutants. The expression of *irrE* mutants was under the control of a GroESL promoter from *D. radiodurans*.

**Figure 2:**
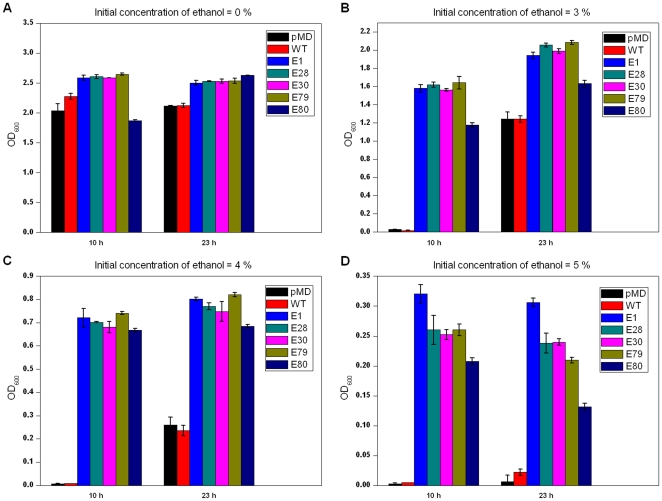
Growth of ethanol-tolerant mutants under ethanol stress. OD_600_ of controls and ethanol-tolerant mutants after cultivation in LB medium supplemented with 0% (**A**), 3% (**B**), 4% (**C**) or 5% (**D**) ethanol for 10 hours and 23 hours. Strains were pMD: strain harboring plasmid pMD18T, WT: strain harboring wild-type *irrE*, E1, E28, E30, E79 and E80: ethanol-tolerant mutants. All data represent the mean values from three independent experiments.

The *irrE* gene was mutated by a standard error-prone PCR protocol (see Materials and Methods). Two sub-libraries, in which the mutation rates were controlled at 1∼3 mutations/kb and 4∼6 mutations/kb, respectively, were constructed and mixed to form a single library. The library was inserted into plasmid pMD18T (**Figure 1**) under the control of the upstream GroESL promoter, and transformed into *E. coli* DH5α cells. Cells were then screened under various conditions, as specified, for mutants with higher tolerances. As mentioned previously, wild-type *irrE* did not confer obviously higher ethanol tolerance to *E. coli*. After selection in the presence of 5% ethanol, five mutants: E1, E28, E30, E79 and E80, which showed much higher ethanol resistance compared with two types of control cells (cells harboring plasmid pMD18T, and cells harboring plasmid pMG1-*irrE*) were obtained. All of these mutants were confirmed by plasmid rescue and retransformation to exclude the effects of possible spontaneous mutation of the host genomic DNA. The growth of cells harboring these *irrE* mutants and of the two control plasmids was tested in LB medium supplemented with different initial concentrations of ethanol (0%, 3%, 4%, 5%, v/v). All of the cells harboring the *irrE* mutants grew much better in medium supplemented with 3%, 4% and 5% ethanol compared to the control cells, which were almost totally inhibited by 5% ethanol. On the other hand, growth of all cells in LB medium with no added ethanol was very similar (**Figure 2**). Slight differences in growth were observed between different ethanol-tolerant mutants of *irrE*, which reflected different ethanol resistance levels for different mutants. Fold increase of OD_600_ at each ethanol concentration was calculated by dividing the OD_600_ value of the mutant cell culture by the OD_600_ value of the control culture harboring the starting plasmid. With increasing ethanol concentration, the fold increase in OD_600_ after 23 hours for the ethanol-tolerant mutants increased. Taking the mutant E1 as an example, the fold increase in OD_600_ values relative to the pMD18T control values were approximately: 1.6-fold in 3% ethanol, 3-fold in 4% ethanol and 48-fold in 5% ethanol.

**Figure 3:**
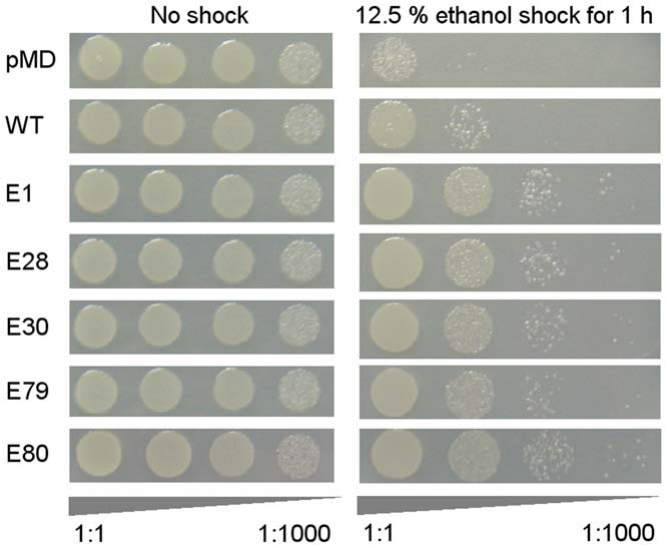
Ethanol shock experiment of ethanol-tolerant mutants. The viabilities of the strains were tested after shocking with 12.5% ethanol for 1 hour (right panel) or without shock (left panel). Strains were pMD: strain harboring plasmid pMD18T, WT: strain harboring wild-type *irrE*, E1, E28, E30, E79 and E80: ethanol-tolerant mutants. Triangles below each panel indicate tenfold serial dilutions of plated cells (1∶1 to 1∶1000, from left to right).

Ethanol shock experiments were subsequently performed to test the tolerance of ethanol-tolerant mutants toward ethanol at a higher concentration, and the results are shown in **Figure 3**. After shocking with 12.5% ethanol for 1 hour, over 10-fold more cells harboring the ethanol-tolerant mutants survived compared with cells harboring wild-type *irrE*, and nearly 100-fold more cells survived compared with cells harboring the starting plasmid pMD18T. These results indicate that not only do the ethanol-tolerant mutants grow better in ethanol of moderate concentrations, but also that they survive much better in ethanol at high concentrations.

### Selection and characterization of butanol-tolerant mutants

**Figure 4:**
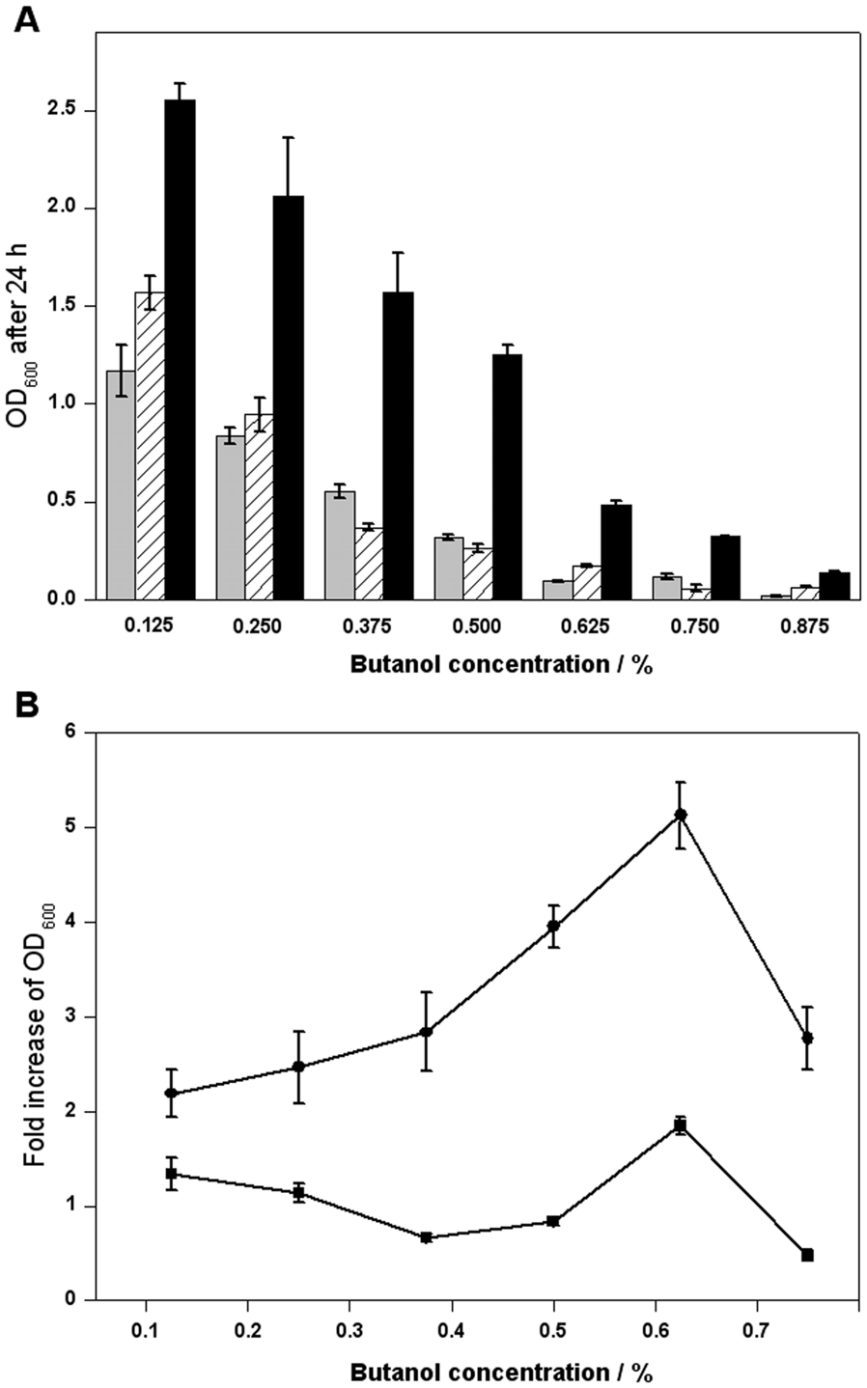
Growth of strains in LB medium supplemented with butanol. **A:** OD_600_ of the strain harboring plasmid pMD18T (grey), wild-type (diagonal) and butanol-tolerant mutant B29 (black) after cultivation in LB medium supplemented with butanol of different concentrations for 24 hours. **B:** Fold increase in OD_600_ for wild-type (filled squares) and mutant B29 (filled circles) compared with control at different butanol concentrations. All data represent the mean values from three independent experiments.

Butanol is another promising biofuel that can be produced by *E. coli*, but it is very toxic to the host cells. Once again, wild-type *irrE* did not confer higher butanol tolerance in *E. coli*. After selection for butanol tolerance as described, one mutant, B29, was isolated and confirmed by retransformation. The growth of cells harboring B29, and the two types of control cells was tested in LB medium supplemented with butanol of different concentrations. The growth advantage of B29 was obvious at all butanol concentrations (**Figure 4A**). As can be seen in **Figure 4B**, the fold increase in OD_600_ values for cells harboring wild-type *irrE* gene are around 1, while those for mutant B29 are over 2 at all concentrations, reaching a maximum of about 5 at the butanol concentration of 0.625%.

**Figure 5:**
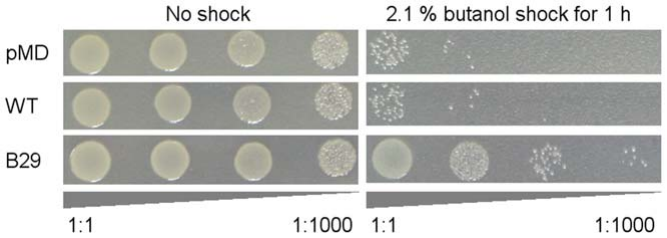
Butanol shock experiment of butanol-tolerant mutant. The viabilities of the strains were tested after shocking with 2.1% butanol for 1 hour (right panel) or without shock (left panel). Strains were pMD: strain harboring plasmid pMD18T, WT: strain harboring wild-type *irrE*, B29: butanol-tolerant mutant. Triangles below each panel indicate tenfold serial dilutions of plated cells (1∶1 to 1∶1000, from left to right).

Butanol shock experiments were performed similarly to the ethanol shock experiments to test the tolerance of mutant B29 towards butanol at a higher concentration. After shocking with 2.1% butanol for 1 hour, most cells harboring wild-type *irrE* or the starting plasmid were killed, while the survival of mutant B29 cells was approximately 100-fold higher (**Figure 5**).

**Figure 6:**
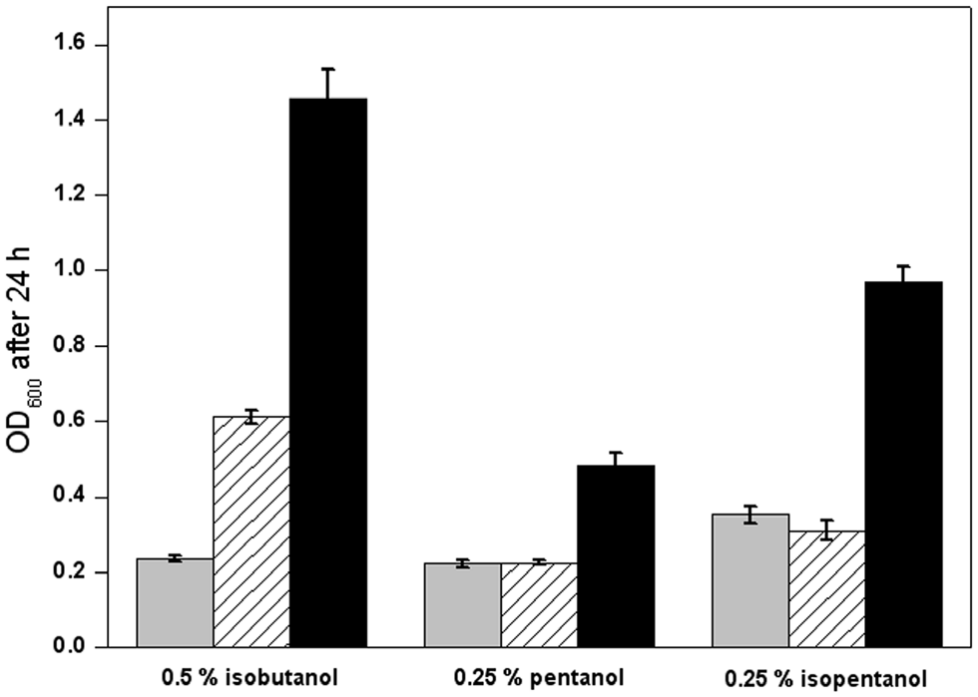
Tolerances of butanol-tolerant mutant toward other C4 or C5 alcohols. OD_600_ of the strain harboring plasmid pMD18T (grey), wild-type (diagonal) and butanol-tolerant mutant B29 (black) was assayed after cultivation in LB medium supplemented with 0.5% isobutanol, 0.25% pentanol or 0.25% isopentanol for 24 hours. All data represent the mean values from three independent experiments.

Besides butanol, other high-grade alcohols with four or five carbon atoms, such as isobutanol, pentanol and isopentanol, can also be used as fuel substitutes, and these alcohols have already been successfully synthesized in engineered *E. coli* cells, albeit at very low concentrations [24]. Because these alcohols have similar molecular compositions and structures to butanol, we suspected that the butanol-tolerant *irrE* mutant B29 should also show tolerance toward these alcohols. As shown in **Figure 6**, B29 indeed showed significantly improved growth in all three high-grade alcohols, compared with control cells harboring wild-type *irrE* or the starting plasmid pMD18T. Interestingly, wild-type *irrE* also conferred a significant degree of tolerance to 0.5% isobutanol, while for 0.5% butanol much less tolerance was seen (**Figure 4A**). It is also noteworthy that pentanol or isopentanol inhibited the growth of B29 cells more intensively than butanol or isobutanol, indicating that these C5 alcohols are much more toxic to B29 cells than the two C4 alcohols.

### Selection and characterization of acetate-tolerant mutants

**Figure 7:**
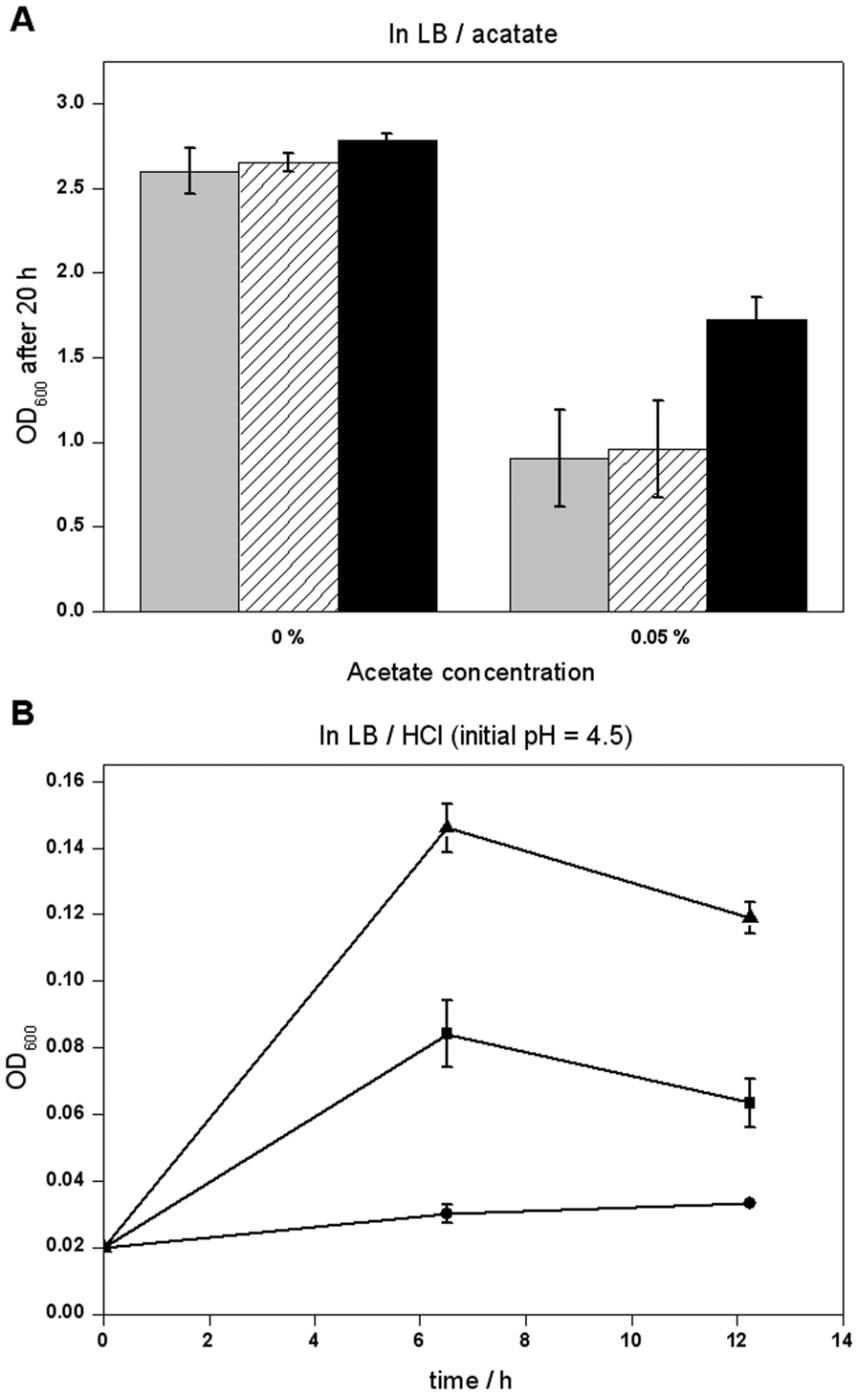
Growth of acetate-tolerant mutant in acids. **A:** OD_600_ of the strain harboring pMD18T (grey), wild-type (diagonal) and acetate-tolerant mutant A15 (black) after cultivation in LB medium supplemented with 0% or 0.05% acetate for 20 hours. **B:** The growth of the strain harboring plasmid pMD18T (filled squares), wild-type (filled circles) and acetate-tolerant mutant A15 (filled triangles) in acidic LB medium (initial pH = 4.5, adjusted with HCl solution).

Acetate is generated during most fermentation processes, and can intensively inhibit cell growth and decrease the yields of target products. Thus, we also attempted to improve acetate tolerance through evolution of *irrE*. After selection for acetate tolerant mutants as described in Materials and Methods, mutant A15, which had the highest acetate resistance, was obtained and confirmed by retransformation. The growth of cells harboring acetate-tolerant mutant A15 and the two control cells was tested in LB medium supplemented with acetate (if applicable). In LB medium, the growth of all the strains was similar, while in LB medium supplemented with 0.05% acetate, the mutant A15 cells had an obvious growth advantage (**Figure 7A**). To test if A15 was also tolerant to low pH caused by inorganic acid, the growth of the strains was assayed in LB medium acidified with HCl (initial pH 4.5). Again, the growth advantage of mutant A15 was significant compared with the two control cells (**Figure 7B**). This demonstrates that the mutant selected for higher tolerance of acetate might also have higher tolerance toward low pH caused by inorganic acid. It is interesting to note that the growth of cells harboring wild-type *irrE* was even worse than that of cells harboring plasmid pMD18T.

### Intracellular reactive oxygen species (ROS) assay of ethanol and butanol mutants

**Figure 8:**
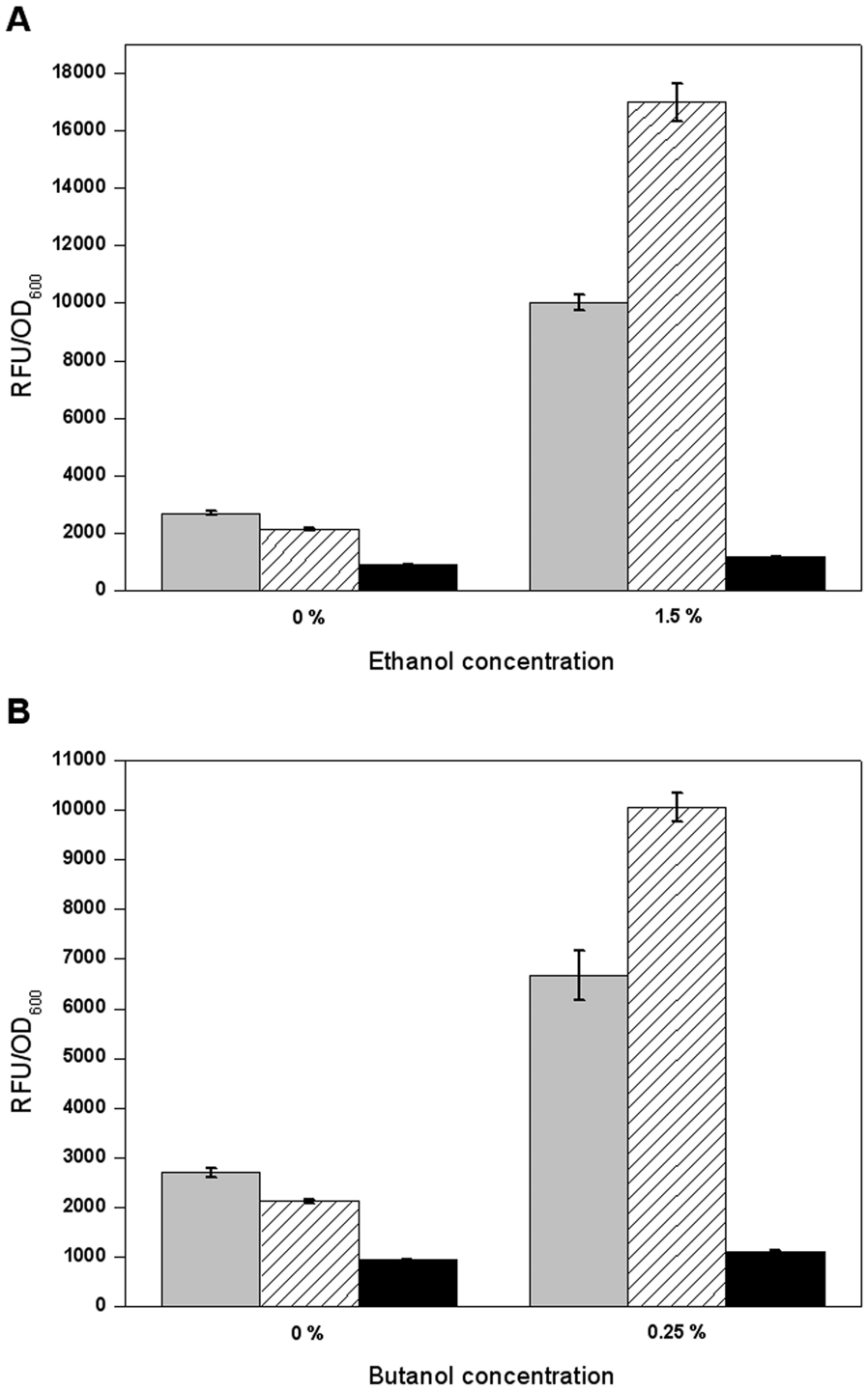
Intracellular ROS assay of alcohol-tolerant mutants. **A:** Relative fluorescence unit (RFU) per OD of cells of the strain harboring plasmid pMD18T (grey), wild-type (diagonal) and ethanol-tolerant mutant E1 (black) cultivated in LB medium supplemented with 0% or 1.5% ethanol. **B:** RFU per OD of cells of the strain harboring plasmid pMD18T (grey), wild-type (diagonal) and butanol-tolerant mutant B29 (black) cultivated in LB medium supplemented with 0% or 0.25% butanol. The cells were dyed with H_2_DCFDA before the RFU assay, as described. All assays were performed in triplicate.

In various microorganisms the intracellular ROS level increases significantly when the cells are challenged by oxidative stress or toxic substances, including butanol [25], [26], [27], [28], [29], indicating that ROS can be used as a indicator of cellular intolerance toward toxic compounds. To test if the *irrE* mutants that conferred higher alcohol tolerances could decrease intracellular ROS levels in *E. coli* cells under alcohol stress, we performed ROS assays with two representative alcohol resistant mutants of *irrE*: ethanol-tolerant mutant E1 and butanol-tolerant mutant B29. To obtain enough cells for the assay, relatively low concentrations of ethanol (1.5%) and butanol (0.25%) were used in the cell culture. After cultivation in the presence or absence of ethanol or butanol, respectively, cells were dyed with a commonly used ROS probe, H_2_DCFDA, which can diffuse into cells and be oxidized by intracellular ROS to generate a fluorescent product. As shown in **Figure 8A**, when there was no ethanol in the medium, the relative fluorescence units (RFU) per cell OD, which reflected average intracellular ROS levels, of all strains was at low levels, and the value for mutant E1 was slightly lower compared to that of the two control cells. When the cells were challenged with 1.5% ethanol, the RFU per cell OD for the two control strains increased significantly, with a higher increase in the strain harboring wild-type *irrE* compared to that in the strain harboring plasmid pMD18T. In sharp contrast, the RFU per OD of cells for mutant E1 in the presence of ethanol only increased slightly (only about 53%), and was still lower than that for the control strain harboring plasmid pMD18T in 0% ethanol. The results for butanol challenges were very similar to those for ethanol (**Figure 8B**). Taken together, we conclude that the *irrE* alcohol-tolerant mutants protect cells, in part, by reducing intracellular ROS levels and thus oxidative damage.

### Sequence alignment and mutational analysis of the mutants

**Figure 9:**
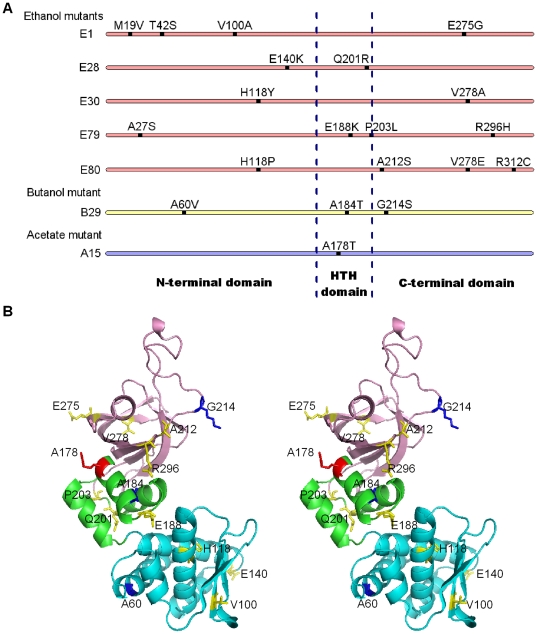
Summary and location of mutation sites. **A:** Summary of mutation sites in the amino acid sequence of all the mutants. The full sequence is divided into three parts by two dashed lines. Each part represents a separate domain of irrE (from left to right: N-terminal domain, HTH domain, C-terminal domain). **B:** Location of mutation sites of all the mutants in a modeled structure of irrE from *D. radiodurans*. Domains of irrE: N-terminal domain, cyan; HTH domain, green; C-terminal domain, pink. Mutation sites of: ethanol-tolerant mutants, yellow; butanol-tolerant mutant, blue; acetate-tolerant mutant, red.

The structure of irrE from *Deinococcus deserti* is known [30] and this protein shares a sequence identity of 64% with irrE from *D. radiodurans*. Three domains are identified in *D. deserti* irrE: the N-terminal domain with a mono zinc metallopeptidase fold, which is speculated to be a peptidase capable of cleaving transcriptional messengers; the middle domain containing a helix-turn-helix (HTH) motif, which is thought to be involved in DNA binding and recognition; the C-terminal domain, which shares a high structural similarity to the GAF domain found in many signal-transduction proteins and which often functions as a sensor by interacting with small molecules [31], [32]. Based on the sequence alignment of these two homologous proteins, we determined the domain boundaries of *D. radiodurans* irrE as follows: the N-terminal domain, residues 1–161; the middle domain, residues 162–203; and the C-terminal domain, residues 204–328 (**Figure 9A**). A homology structural model for *D. radiodurans* irrE (residues 44–303) was also constructed using SWISS-MODEL, with irrE from *D. deserti* as a template. The structure stereoview of *D. radiodurans* irrE was then prepared with PyMOL (**Figure 9B**). *D. radiodurans* irrE has 43 extra residues at the N-terminus and 24 extra residues at the C-terminus compared with the solved structure of *D. deserti* irrE; therefore, these extra residues are omitted in the homology model. The irrE mutants obtained in this study were sequenced, and the mutations are summarized at both the primary sequence level (for all mutations), and at the homology model level (for mutations within the range of residues 44–303), shown in **Figure 9A** and **Figure 9B**.

## Discussion

We have developed a novel method to increase strain tolerances towards different stresses by introducing mutants of an exogenous global regulator, *irrE* from *D. radiodurans*, into target cells. *E. coli*, which is widely used for the production of bio-fuels and other chemicals, was chosen as the model strain. Mutants were engineered to investigate three meaningful phenotypes, *i.e.*, tolerances toward ethanol, butanol and acetate. This method supplements the existing methods for tolerance improvement of industrial strains, for example gTME.

Wild-type *D. radiodurans irrE* has been shown to increase the resistance of *E. coli* toward radiation, osmotic pressure, heat stress and oxidative stress [16], [23]. These findings show that an exogenous global regulator could function well in a non-native host, and that there is commonality between the regulation systems of *D. radiodurans* and *E. coli*. Therefore it is not surprising that the *irrE* mutants that we obtained through random mutagenesis and selection confer significantly higher tolerances toward alcohols and acetate in *E. coli*, although wild-type *irrE* only slightly improved tolerances towards ethanol and acid in *Zymomonas mobilis* [33], and is not able to measurably enhance tolerances toward these stresses in *E. coli*.

Ethanol is one of the most important bio-fuels, and the production of ethanol using *E. coli* has been explored extensively [34], [35], [36], [37]. The ethanol-tolerant *irrE* mutants we obtained will be useful for the design of ethanol-producing strains. For example, two of our *irrE* mutants enable cells to grow to an OD value of more than 2 in the presence of 3% ethanol after 23 hours, which is almost the same as the control strains in the absence of ethanol (see **Figure 2B**).

Butanol is another promising fuel substitute [38], and traditionally *Clostridium acetobutylicum* has been employed as a workhorse, but increasingly *E. coli* is being explored for butanol production [24], [38]. However, butanol is very toxic to *E. coli* cells, and the concentrations of butanol achieved using *E. coli* are often under 0.1%, compared to nearly 2% obtained for *Clostridium acetobutylicum* [5]. The butanol-tolerant *irrE* mutant we obtained has significant growth advantages in the presence of 0.125% to 0.875% butanol (see **Figure 4**), and was able to reach an OD value of more than 2 in the presence of 0.125% and 0.25% butanol, similar to that of the control strains in the absence of butanol. We have also shown that the butanol-tolerant mutant has significantly higher tolerances towards three other kinds of high-grade alcohols, and this indicates how tolerances among these alcohols are related. This cross-tolerance towards different high-grade alcohols will be meaningful when two or more high-grade alcohols are simultaneously produced during a single fermentation process [39], [40].

The inhibition effect of carboxylic acids on cell growth is, in part, attributed to low pH [25]. We have shown that the acetate-tolerant mutant A15 had higher tolerance towards not just acetate, but also low pH values caused by inorganic acid (see **Figure 7**). The high tolerance of A15 towards low pH might be part of the reason for its higher tolerance toward acetate.

The putative domains of irrE from *D. radiodurans* were determined by sequence alignment with irrE from *D. deserti* as described (see **Figure 9**). It is noteworthy that the mutations contained in the mutants we obtained occur in all three domains, which are postulated to exhibit protease, DNA recognition, and small molecule sensoring functions, respectively. This implies that the improvements in the phenotypes we engineered were achieved through multiple mechanisms. Ethanol-tolerant mutants E1, E28, E30, E79 and E80 contain four, two, two, four and four amino acid mutations in the three domains, respectively. Butanol-tolerant mutant B29 has three missense mutations: A60V, A184T, and G214S, one in each domain. Acetate-tolerant mutant A15 has one missense mutation, A178T, in the middle domain or HTH domain. It is worth noting that the mutants E30 and E80 contain mutations at two common sites: H118 and V278. Of these two mutations, H118 corresponds to H82 of *D. deserti* irrE according to sequence alignment, and is directly involved in the zinc-binding function of the N-terminal domain. The mutation at this residue presumably affects the putative peptidase activity of the N-terminal domain directly. The mutational effects of other amino acid changes, however, are less straightforward, in part because the regulation mechanism of irrE itself has not been elucidated. Transcriptomic and proteomic analyses are being performed on cells containing representative *irrE* tolerance mutants to dissect how transcriptional and expression profiles are reprogrammed in these cells.

This work has clearly shown that cellular tolerances toward stresses can be improved by introducing engineered exogenous global regulators into an industrially relevant strain. To our knowledge, this is the first time that an exogenous global regulator has been artificially evolved to suit its new host. The application of wild-type or engineered exogenous global regulators can be combined with other current methods (*e.g.*, gTME) to yield better strain improvements. Tolerance improvement using global approaches seems to be restricted by the fact that the recognition sequences and regulation functions of transcriptional factors are limited, so the alteration of transcriptional profiles through engineering of these factors is also limited. For example, in the application of gTME in *E. coli* for enhancement of ethanol tolerance, the number of genes altered by mutated sigma factors (around one hundred) is much less than the number of genes responding to ethanol stress (more than three hundred) [14]. Similarly, ribosome engineering will only interfere with the protein translation process [10], [41], [42]. However, cellular tolerances are likely to require coordinated responses at transcriptomic, proteomic and post-translation regulation levels [43], [44]. Introducing and engineering exogenous global regulators, which may have different recognition sequences and regulation mechanisms, would extend the perturbation range of transcriptional profiles, and may lead to larger improvements of strain tolerances. Moreover, engineering of global regulators that have multiple functions (as in the case of irrE) may provide ways to simultaneously make alterations at the transcriptomic, proteomic, and perhaps post-translation regulation levels, and thus lead to rapid and significant improvements in cellular tolerances.

## Materials and Methods

### Materials

Restriction enzymes and DNA-modifying enzymes were purchased from New England Biolabs (Beverly, MA, USA). LA Taq DNA polymerase was purchased from TaKaRa (Dalian, China). Oligonucleotides were synthesized by Invitrogen (Carlsbad, CA, USA). Sequence analysis was performed either by Sunbiotech (Beijing, China) or by Invitrogen. The kits for DNA purification, gel recovery and plasmid mini-prep were from either Tiangen (Beijing, China) or QIAgen (Valencia, CA, USA). *E. coli* strain DH5α and pMD18T was from TaKaRa. 2′,7′–dichlorofluorescein diacetate (H_2_DCFDA) for the intracellular ROS assay was from Sigma-Aldrich (St. Louis, MI, USA).

### Library Construction and Selection

Error-prone PCR was employed to construct the library. The *irrE* gene and the upstream groESL promoter were amplified from pMG1-*irrE* [23], using primers 5′-GAGATCTATCGATGCATGCCATGGT-3′ and 5′-TCCCCGCGGAGATCTCCAGTTCAC-3′. A standard error-prone PCR protocol was employed for library construction, and the mutation rate was controlled by adjusting the concentration of manganese ions. The products of error-prone PCR were purified, digested and inserted into *Nco*I and *Sac*II sites of plasmid pMG1-*irrE* replacing the wild-type *irrE* gene and GroESL promoter (**Figure 1**). The ligation products were transformed into *E. coli* DH5α competent cells. Cells were plated on LB-agar plates containing 50 µg/ml ampicillin, incubated at 37°C overnight, and scraped off to create a liquid library. The library size was approximately 10^6^.

The liquid library was inoculated into challenging medium to select for mutants of higher tolerances toward different stresses. Different concentrations of challenging substances were tested and concentrations that allowed growth of the liquid library were then used for selection. For selection of ethanol tolerance, LB medium containing 5% (v/v) ethanol and 50 µg/ml ampicillin was used as a challenging medium. For selection of butanol tolerance, LB medium containing 0.75% (v/v) butanol and 50 µg/ml ampicillin was used. For selection of acetate tolerance, LB medium containing 0.05% (v/v) acetate and 50 µg/ml ampicillin was used. The library was grown at 37°C in challenging mediums, subcultured twice and then plated on LB-agar plates containing 50 µg/ml ampicillin and incubated at 37°C overnight. For each tolerance test, about 50 single colonies were then picked for growth assay in challenging medium. The plasmids of the mutants with significantly higher tolerances were rescued and retransformed into fresh *E. coli* DH5α cells, and the growth in challenging media was retested, to separate the effects of the mutated *irrE* gene from any possible spontaneous chromosomal mutations acquired during the selection process.

### Growth assay

To test the growth of mutants in challenging media, *E. coli* DH5α cells harboring plasmids pMD18T and pMG1-*irrE* (wild-type), and the mutants with higher tolerances toward different stresses were grown overnight at 37°C in LB medium containing 50 µg/ml ampicillin. The overnight cultures were diluted to an OD_600_ of 0.02 in LB medium containing 50 µg/ml ampicillin and challenging substances of different concentrations, and then grown at 37°C. OD_600_ was monitored during growth. The growth of butanol-tolerant mutant B29 in other high-grade alcohols was also tested. For acetate-tolerant mutant A15, growth in LB medium supplemented with inorganic acid HCl to produce a lower pH (4.5) was also tested.

### Shock experiments

To test the tolerance of ethanol-tolerant and butanol-tolerant mutants toward extremely high alcohol stresses, shock experiments were performed with much higher concentrations of ethanol and butanol. Cells were grown overnight at 37°C in LB medium containing 50 µg/ml ampicillin, then diluted at a ratio of 1∶100 into fresh LB medium containing 50 µg/ml ampicillin, and grown at 37°C to logarithmic phase with an OD_600_ of 0.5∼1.0. The cultures were then diluted with fresh LB medium to an OD_600_ of 0.5, and ethanol or butanol was added to final concentrations of 12.5% (v/v) and 2.1% (v/v), respectively. After incubation at 37°C for 1 h, the cultures were serially diluted, plated onto LB/agar plates and incubated at 37°C overnight for 24 hours and then photographed.

### ROS assay

Cells were grown at 37°C in LB medium overnight, then diluted at a ratio of 1∶100 into fresh LB medium, or LB medium containing 1.5% ethanol or 0.25% butanol and grown at 37°C overnight. All media used were supplemented with 50 µg ml^−1^ ampicillin. The cultures were then centrifuged, and the cell pellets were washed and resuspended with phosphate buffered saline (PBS) pH 7.0. For each sample, 100 µL of cell suspension was then transferred into a well of a 96-well plate (black background), and diluted with 95 µL PBS buffer. 5 µL 2.5 mg/ml H_2_DCFDA solution was added into each well, and the plate was incubated at 37°C for 45 minutes. The fluorescence intensity was then assayed with a Spectra-Max M2 microtiter reader (Molecular Devices, Sunnyvale, CA, USA) (excitation, 490 nm; emission, 519 nm). The OD_600_ of the cell suspension was also assayed.
